# On gender and cognitive flexibility. The REM-ACT study: Acceptance and commitment therapy versus a mindfulness-based emotional regulation intervention in anxiety disorders. A randomized controlled trial

**DOI:** 10.1192/j.eurpsy.2021.2080

**Published:** 2021-08-13

**Authors:** E. Fernández-Jiménez, E. Vidal-Bermejo, I. Torrea-Araiz, T. Castellanos-Villaverde, G. Navarro-Oliver, A. Hospital-Moreno

**Affiliations:** 1 Idipaz, Department Of Psychiatry, Clinical Psychology And Mental Health, La Paz University Hospital, Madrid, Spain; 2 Department Of Psychiatry, Clinical Psychology And Mental Health, La Paz University Hospital, Madrid, Spain

**Keywords:** Mindfulness-based Emotional Regulation, acceptance and commitment therapy, randomized controlled trial, cognitive flexibility

## Abstract

**Introduction:**

Research is needed to explore whether cognitive flexibility may account for potential gender differences after mindfulness-based interventions.

**Objectives:**

To compare the effectiveness of Acceptance and Commitment Therapy (ACT) versus a Mindfulness-based Emotional Regulation (MER) intervention on cognitive flexibility according to gender.

**Methods:**

This study was carried out in a Mental Health Unit in Spain (Colmenar Viejo, Madrid). Firstly, 80 adult patients with anxiety disorders were randomized according to the score on the Acceptance and Action Questionnaire-II (blocking factor), of whom, 64 patients decided to participate (mean age = 40.66, S.D. = 11.43; 40 females). Each intervention was weekly, during 8 weeks, guided by two Clinical Psychology residents. A 2x2x2 mixed ANOVA (pre-post change x intervention type x gender) was conducted, with Sidak-correction post hoc tests. The dependent variable was the score on TMT-B.

**Results:**

A natural logarithmic transformation was conducted to correct violation of normality and homoscedasticity assumptions. No statistically significant differences were observed on age or gender between interventions. No statistically significant interaction effect was observed between pre-post change x intervention x gender [F_(1, 52)_ = .014, p = .907]. An interaction effect was observed between pre-post change x intervention [F_(1, 52)_ = 4.180, p = .046; statistical power observed = 52%]: while TMT-B improved after ACT (p = .001; Cohen’s d = 0.607), there were no changes after MER (p = .367; Cohen’s d = 0.097).

**Conclusions:**

These medium effect-size results confirm previous findings of our research team indicating cognitive flexibility improves after ACT but not after MER.

**Disclosure:**

No significant relationships.

